# The Mediation Role of Socioeconomic Status on Racial and Ethnic Differences in Advance Care Planning

**DOI:** 10.1093/geroni/igaa057.1068

**Published:** 2020-12-16

**Authors:** Junghee Han, Taekbeen Nam

**Affiliations:** 1 Calvin University, Grand Rapids, Michigan, United States; 2 Grand Rapids, Michigan, United States

## Abstract

Background. Most research on EOL care planning has focused on racial/ethnic differences in completing advanced directives (AD) rather than the pathways of the disparities. Therefore, this study aims to examine the mediating role of education and income in racial/ethnic differences in EOL care planning. Methods. A secondary data analysis of Health and Retirements Study (HRS) 2004-2014 wave was used. The sample included 6,518 participants ((≥ 65 years old). The independent variable measured the respondents’ race and ethnicity and the dependent variable measured the completion rate of living wills or the Durable Power of Attorney of Health Care (DPAHC). Covariates included gender, age, marital status, religion, place f birth, educational attainment, income, cognitive function, limitations in physical functioning, geriatric syndromes, and the number of progressive chronic disease. Results. The hierarchical logistic regression analysis showed that race/ethnicity was a significant predictor of completing AD (p≤0.001). Mediation analysis, Karlson, Home, and Breen (KHB), revealed that both education and income explained 14.4% of racial/ethnic differences in completion of living wills for non-Hispanic Blacks and 17.6% for Hispanics. Similarly, education and income accounted for 17.6% of racial/ethnic disparities in completion of DPAHC for non-Hispanic Blacks and 20.9% for Hispanics. In particular, education had stronger mediating effect on the outcome variables than income. Discussion. The findings suggest the importance of targeted educational interventions for people of color with lower SES to raise their awareness of benefits of advance care planning and increase their access to higher quality of EOL care.

